# A temporal coupling hypothesis for chemoresistance in canine DLBCL and osteosarcoma: stress signalling and microRNA regulation

**DOI:** 10.1016/j.vas.2026.100916

**Published:** 2026-10-23

**Authors:** Soroush Akbari-Ardabili

**Affiliations:** a Division of Biochemistry, Department of Basic Sciences, Faculty of Veterinary Medicine, Urmia University, Urmia, Iran; b R&D Department, Rahavard Afarinesh Kavir Company, Qom, Iran

**Keywords:** Canine cancer, Chemoresistance, Diffuse large B-cell lymphoma, Osteosarcoma, microRNA, Hippo–YAP pathway, Temporal dynamics

## Abstract

Acquired chemoresistance remains a major clinical problem in canine diffuse large B-cell lymphoma (DLBCL) and osteosarcoma. Existing canine studies identify established resistance-associated features, including drug efflux, altered apoptosis, DNA repair, epigenetic change, and microRNA (miRNA) dysregulation, and support selected stress-response components as biologically relevant. However, the available canine evidence does not establish a chemotherapy-induced temporal sequence linking early stress signalling to later miRNA regulation. Likewise, the YAP–DDX17–Microprocessor relationship is supported by mechanistic evidence from mammalian/human systems but has not been demonstrated as a chemotherapy-induced coupling mechanism in canine tumours. This Opinion Paper therefore presents a testable temporal coupling hypothesis rather than a validated canine mechanism. The hypothesis asks whether early treatment-induced stress responses reproducibly precede and are associated with later miRNA regulation, and whether any such relationship reflects mechanistic dependence. YAP–DDX17–Microprocessor is considered one experimentally tractable but unvalidated candidate bridge, not a required component of the broader hypothesis. The article distinguishes established canine evidence from cross-species evidence and theoretical inference, and outlines the longitudinal and perturbational studies needed to test the hypothesis while accounting for pharmacokinetics, miRNA biogenesis, and tumour-microenvironment effects before clinical translation.

## Highlights

• Canine chemoresistance is reframed as a time-dependent process.
• Static resistance markers may miss early adaptive transitions.
• Stress signalling and microRNA regulation form testable temporal windows.
• One molecular bridge is proposed but remains unvalidated in canine tumours.
• Validation requires time-course experiments and cell-fate measurements.

## 1 Introduction: temporal questions in canine chemoresistance

Acquired chemoresistance remains an important limitation in the treatment of canine lymphoma and osteosarcoma. In primary nodal DLBCL and related peripheral nodal B-cell lymphoma cohorts, CHOP-based chemotherapy produces high initial response rates, but remissions are often finite and disease progression remains common (Childress et al., 2018; Davies et al., 2018; Hawkes et al., 2024; Vos et al., 2019). In canine osteosarcoma, local tumour control combined with adjuvant chemotherapy improves survival relative to local control alone, yet metastatic progression remains common and long-term survival is uncommon (Berg et al., 1997; Selvarajah & Kirpensteijn, 2010). These disease-specific clinical patterns establish that meaningful initial treatment responses can be followed by later progression, but they do not identify the molecular sequence responsible for treatment failure. They therefore motivate, rather than demonstrate, the temporal hypothesis developed here.

Most molecular studies of chemoresistance have understandably focused on the resistant state itself. This approach has identified important mechanisms, including ATP-binding cassette drug efflux, altered apoptotic signalling, enhanced DNA repair, epigenetic change, and microRNA (miRNA) dysregulation (Craig et al., 2019; Elshafie et al., 2023; Elshafie et al., 2024; Klopfleisch et al., 2016; Packeiser et al., 2023; Zandvliet & Teske, 2015; Zorzan et al., 2022). These mechanisms are biologically and clinically relevant, but they often describe the molecular phenotype of established resistance. They do not always explain how an initially responsive tumour moves towards relapse, or which early post-treatment events determine whether a tumour follows a sensitive or resistant trajectory.

This temporal dimension remains poorly resolved. A diagnostic biopsy, a resistant cell line, or a relapsed tumour sample can reveal what resistance looks like after selection has occurred, but these snapshots provide limited information about the sequence of events that unfolded after chemotherapy exposure. In canine oncology, relatively few studies have sampled tumours or model systems densely enough during the first hours and days after treatment to determine whether early stress responses reproducibly precede and are associated with later regulatory changes. As a result, potentially important temporal relationships between stress signalling, miRNA regulation, and cell-fate commitment remain difficult to evaluate.

This Opinion Paper proposes that chemoresistance in canine DLBCL and osteosarcoma be studied not only as a static molecular state but also as a testable sequence linking early treatment-induced stress signalling to later miRNA-mediated fate regulation. The novelty does not lie in identifying individual stress pathways or miRNAs as resistance-associated markers. Rather, it lies in testing whether their relative timing and functional dependence reveal how an initially responsive tumour moves towards persistence or relapse. Here, “temporal coupling failure” refers to loss or mistiming of this proposed coordination between the early and delayed responses, rather than simply an abnormal level of either marker class. The model is intended as a working hypothesis, not an established canine paradigm. Its value depends on whether it generates falsifiable predictions while clearly distinguishing canine evidence from cross-species inference.

## 2 Static resistance markers are necessary but temporally incomplete

Established resistance mechanisms should not be dismissed. Drug efflux through ATP-binding cassette transporters, altered apoptotic control, DNA repair capacity, epigenetic regulation, and changes in non-coding RNA networks all contribute to the behaviour of resistant cancer cells (Craig et al., 2019; Elshafie et al., 2023; Elshafie et al., 2024; Klopfleisch et al., 2016; Packeiser et al., 2023; Zandvliet & Teske, 2015; Zorzan et al., 2022). In canine oncology, these mechanisms have provided an essential vocabulary for describing why a tumour may become less responsive to chemotherapy. They also remain clinically relevant, because relapsed tumours and drug-resistant cell lines often display stable molecular features that can plausibly support survival under treatment pressure.

The limitation is not that these markers are unimportant, but that they are usually measured after resistance is already established. A relapsed lymph node biopsy, a diagnostic sample associated with poor outcome, or an experimentally selected resistant cell line can identify molecules enriched in resistant disease. However, these approaches have limited ability to resolve the early sequence of events that occurs after chemotherapy exposure. They rarely show whether resistance begins with an inadequate stress response, a failure to sustain tumour-suppressive regulation, selection of a pre-existing subclone, or some combination of these processes.

This distinction is important for canine diffuse large B-cell lymphoma. Several studies have examined gene-expression or miRNA patterns associated with relapse, drug resistance, or early treatment failure in canine lymphoma (Craig et al., 2019; Elshafie et al., 2023; Elshafie et al., 2024; Tani et al., 2021; Toyoda et al., 2024; Zandvliet et al., 2014). These studies are valuable because they identify candidate pathways and biomarkers that may be linked to outcome. Yet most of them are not designed to ask whether early post-treatment signalling changes are temporally connected to later miRNA shifts. As a result, miRNAs may be interpreted as static biomarkers of prognosis or relapse, rather than as potential components of a time-dependent regulatory response.

A similar caution applies to canine osteosarcoma. Molecular and miRNA studies have implicated tumour-suppressive and oncogenic miRNAs in aggressive behaviour, invasion, and prognosis (Gourbault & Llobat, 2020; Lopez et al., 2018; Ludwig et al., 2024). These findings support the biological relevance of miRNA regulation in canine cancer, but they do not by themselves define when those changes arise after chemotherapy or whether they are causally linked to early stress signalling. Static molecular associations can therefore generate hypotheses about resistance, but they cannot alone establish a temporal mechanism.

The central point of this Opinion Paper is that static and temporal models should be treated as complementary. Static markers describe the molecular architecture of resistant disease; temporal models ask how that architecture is reached. For canine chemoresistance research, the next useful question is not only which markers differ between sensitive and resistant states, but whether early treatment-induced stress responses reproducibly precede and are associated with later regulatory changes. Mechanistic dependence would then require separate perturbational testing.

Canine DLBCL and osteosarcoma are considered together here as complementary test contexts, not as biologically or therapeutically equivalent tumours. DLBCL is considered in the setting of CHOP-based therapy and remission followed by relapse, whereas osteosarcoma is considered in the setting of local control, adjuvant chemotherapy, and metastatic progression (Berg et al., 1997; Childress et al., 2018; Davies et al., 2018; Hawkes et al., 2024; Selvarajah & Kirpensteijn, 2010; Vos et al., 2019). The rationale is therefore comparative. The same temporal question can be tested in two distinct treatment contexts, while the relevant kinetics, molecular readouts, and even the presence of the proposed coupling mechanism may differ by tumour type. Evidence from either tumour type should not be taken as validation in the other or extrapolated to canine cancer generally.

To distinguish direct evidence from cross-species plausibility and hypothesis generation, the evidence discussed below is organised into four levels. First, established canine evidence refers to observations demonstrated directly in canine tumours or dog-derived cancer systems, without implying temporal or causal coupling. Second, mammalian/human evidence refers to mechanisms demonstrated outside canine oncology that establish biological plausibility but not canine-specific treatment kinetics. Third, theoretical inference refers to relationships proposed by integrating otherwise separate evidence streams. Fourth, proposed hypotheses are the specific temporal and causal predictions that require prospective testing in dog-derived systems. Under this hierarchy, current canine evidence supports selected stress-response components and miRNA dysregulation as separate observations, whereas the ordered early-stress-to-delayed-miRNA sequence and the YAP–DDX17–Microprocessor coupling remain theoretical inferences and proposed hypotheses rather than established canine mechanisms.

## 3 A proposed temporal coupling hypothesis

Within these two disease-specific test contexts, the temporal coupling concept is presented as a hypothesis generated from the evidence hierarchy above, not as a mechanism established in canine tumours. It asks whether acquired chemoresistance may involve two broad regulatory windows: an early stress-signalling response and a later miRNA-regulatory response. The hypothesis is not intended to replace established mechanisms of resistance, nor does it imply that all canine tumours follow a single kinetic programme. Its purpose is narrower: to provide a testable way of asking whether the timing and coordination of treatment-induced responses influence whether a tumour cell moves towards death, persistence, or later relapse. This proposed temporal structure, including one candidate YAP–DDX17 mechanistic bridge and the boundary between supported and unvalidated claims, is summarised in Fig. 1.

**Fig. 1:**
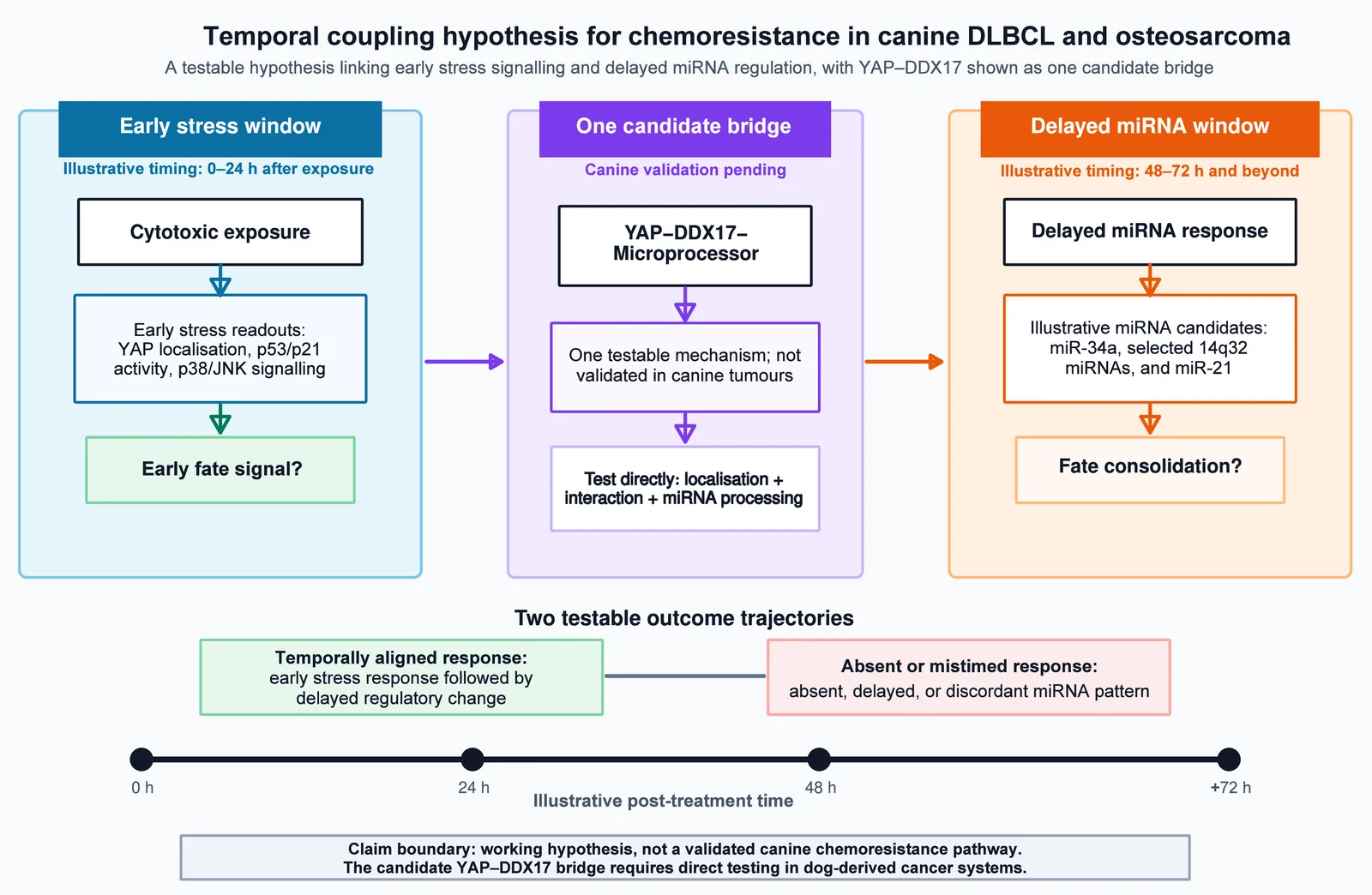
Proposed temporal coupling hypothesis for studying chemoresistance in canine DLBCL and osteosarcoma. The model frames acquired chemoresistance as a possible failure of coordination between an early stress-signalling window and a delayed miRNA-regulatory window after cytotoxic exposure. The 0–24 h and 48–72 h intervals are illustrative working windows rather than validated canine-specific kinetic boundaries; the relevant timing should be empirically calibrated for each tumour and treatment context. Such calibration should also account for drug-specific pharmacokinetics and treatment schedule, because the same clock time after administration may correspond to different exposure and pharmacodynamic states for different agents. Temporal precedence in this scheme represents a testable temporal relationship and should not be interpreted as evidence of mechanistic dependence or causal coupling without perturbational and pathway-specific validation. Candidate early readouts include YAP localisation or phosphorylation, p53/p21 pathway activity, and stress-activated kinase signalling. The YAP–DDX17–Microprocessor axis is shown as one candidate mechanistic bridge between early Hippo–YAP signalling and later miRNA processing, but this mechanism remains unvalidated in canine tumour systems. Delayed miRNA responses should be evaluated as context-dependent regulatory patterns rather than as a binary sensitivity–resistance axis. miR-34a, selected 14q32 miRNAs, and miR-21 are shown only as illustrative candidate readouts; current canine evidence does not establish any of them as directionally specific markers of chemotherapy sensitivity or resistance. Changes in mature miRNA abundance are not considered evidence of altered miRNA biogenesis; the latter requires separate assessment of precursor and mature miRNA species. The schematic emphasises the tumour-cell-intrinsic core of the hypothesis; immune and stromal components of the tumour microenvironment may modify both the early and delayed response phases and should be evaluated separately in appropriate experimental systems. The model is intended to generate falsifiable experimental questions, not to define a validated canine chemoresistance pathway. Abbreviations: DDX17, DEAD-box helicase 17; miRNA, microRNA; YAP, Yes-associated protein.

To avoid conflating temporal order with mechanism, four inferential steps are distinguished. Temporal precedence requires only that an early stress response occur before a later miRNA change. Temporal association requires a reproducible relationship between the early and later responses across repeated observations. Mechanistic dependence requires perturbation of the proposed upstream signalling node to alter the later miRNA response. Causal coupling is a stronger claim and requires convergent evidence that the downstream response is mediated through the proposed pathway rather than merely occurring after, or in parallel with, the upstream response. Thus, temporal precedence or temporal association alone would not be considered evidence of mechanistic or causal coupling. For hypothesis testing, an absent, markedly delayed, or temporally inconsistent downstream miRNA response would be considered evidence against the proposed temporal sequence. A reproducibly ordered response would support temporal association, whereas designation as mechanistically dependent or causally coupled would require the additional perturbational and pathway-specific evidence described below.

The first window is an early stress-signalling response after cytotoxic exposure. In principle, this phase may involve pathways that sense DNA damage, cytoskeletal stress, oxidative stress, or broader loss of cellular homeostasis. Candidate pathways include Hippo–YAP signalling, p53 pathway activity, and p38/c-Jun N-terminal kinase (JNK) stress-activated kinase signalling (Bao et al., 2011; Canovas & Nebreda, 2021; Liu et al., 2021; Lv & Zhou, 2023; Raj & Bam, 2019; Zhang et al., 2009). In canine systems, some elements of this response are better supported than others. For example, p53 pathway activation after DNA-damaging treatment has direct canine experimental support, whereas chemotherapy-induced Hippo–YAP kinetics and p38 timing in canine cancer cells remain more inferential. This distinction matters: conservation of pathway components supports biological plausibility, but it does not establish that the same timing, amplitude, or regulatory consequences occur in canine tumours.

The second window is a delayed miRNA-regulatory response. miRNAs are attractive candidates for this later phase because they can coordinately influence apoptosis, proliferation, invasion, and stress adaptation. In canine lymphoma and osteosarcoma, several studies have linked miRNA expression patterns with prognosis, relapse, aggressive behaviour, or tumour biology, including miR-21, miR-34a, and 14q32-associated miRNAs (Craig et al., 2019; Elshafie et al., 2023; Elshafie et al., 2024; Gourbault & Llobat, 2020; Lopez et al., 2018; Ludwig et al., 2024). These data support the biological relevance of miRNA regulation in canine tumours, but most available studies remain cross-sectional. They do not yet show whether miRNA changes emerge after chemotherapy in a reproducible temporal order or whether they are driven by earlier stress-signalling events. Importantly, changes in mature miRNA abundance should not be equated with altered miRNA biogenesis. Mature miRNA levels reflect the combined effects of transcription, precursor processing, downstream maturation, and RNA turnover; mature-miRNA profiling alone therefore cannot identify which regulatory step produced an observed change. Claims concerning altered biogenesis require direct examination of precursor and mature miRNA species rather than mature miRNA abundance alone (Mori et al., 2014; Wu, 2020; Xu et al., 2022; Yu & Guan, 2014).

The hypothesis therefore considers two candidate response phases rather than a demonstrated regulatory sequence. Cytotoxic therapy may induce an early stress response that temporarily alters the regulatory state of the tumour cell. The theoretical inference is that a later miRNA response may then contribute to consolidation of cell fate. However, any delayed miRNA response is likely to reflect a context-dependent regulatory network rather than a simple balance between tumour-suppressive and pro-survival miRNAs. Its functional consequences may vary with tumour type, treatment, cellular state, and the combined activity of multiple miRNAs and their targets. Candidate miRNAs such as miR-34a, selected 14q32 miRNAs, and miR-21 are therefore presented only as illustrative readouts whose direction and functional significance require empirical testing in each canine treatment context.

This model also reframes how candidate biomarkers should be interpreted. A single baseline measurement of a resistance-associated marker may be clinically useful, but it cannot determine whether an ordered post-treatment relationship occurred, much less whether mechanistic dependence was present. Similarly, a relapsed tumour sample can show the molecular endpoint of selection, but not the sequence that produced it.

## 4 YAP–DDX17–Microprocessor as one candidate mechanistic bridge

The temporal hypothesis does not depend on a single molecular bridge between the early and delayed response phases. YAP–DDX17–Microprocessor is considered here as one experimentally tractable candidate mechanism because mammalian studies provide a plausible route by which Hippo–YAP signalling could influence miRNA biogenesis. It should not be interpreted as a required component of the temporal hypothesis or as an established pathway in canine cancer.

In mammalian cells, YAP localisation is a major output of Hippo pathway activity. When Hippo signalling is inactive, YAP can accumulate in the nucleus and support transcriptional programmes related to growth and survival. When upstream Hippo kinases are activated, YAP is phosphorylated and retained in the cytoplasm, reducing its nuclear activity (Bao et al., 2011; Liu et al., 2021; Lv & Zhou, 2023). Separately, human-cell studies have linked YAP to the regulation of miRNA biogenesis through interaction with DDX17, also known as p72, a DEAD-box RNA helicase that contributes to Microprocessor function during pri-miRNA processing (Mori et al., 2014; Wu, 2020; Xu et al., 2022; Yu & Guan, 2014). This provides a biologically plausible bridge between YAP localisation and later changes in mature miRNA abundance.

However, altered mature miRNA abundance is not itself evidence of altered Microprocessor activity. The proposed YAP–DDX17 mechanism concerns pri-to-pre-miRNA processing specifically. A mechanistic test must therefore distinguish changes in miRNA transcription from changes in precursor processing by measuring pri-miRNA, pre-miRNA, and mature miRNA species in parallel. A treatment-associated change in pri-to-pre processing accompanied by a corresponding downstream change in mature miRNA abundance would be more consistent with altered Microprocessor processing, whereas a change confined to mature miRNA abundance would not establish this mechanism. These patterns should still be interpreted cautiously because precursor stability and downstream processing can also affect RNA abundance.

The relevance of this mechanism to canine chemoresistance remains unresolved. The core Hippo pathway is conserved, and DDX17/Microprocessor biology is broadly conserved across mammals, but conservation alone does not prove that chemotherapy induces the same YAP localisation dynamics or YAP–DDX17 interaction in canine tumour cells. Nor does it prove that any such interaction controls the timing or direction of miRNA changes after treatment. These are empirical questions.

## 5 What evidence would support or refute the hypothesis?

The value of the proposed hypothesis depends on whether increasingly strong levels of inference can be established experimentally. Temporal precedence and association should be demonstrated before mechanistic dependence is tested, while causal coupling should be reserved for evidence linking the upstream and downstream responses through the proposed mechanism. A later clinical layer may be considered only after these cellular and assay-level foundations are established. At the experimental level, drug exposure and sampling schedules should be justified by canine pharmacokinetic context where available, or by pilot dose–response experiments that leave enough viable cells to study adaptive responses rather than only acute cytotoxic collapse.

Pharmacokinetic context is particularly important because clock time after drug administration is not equivalent to biological exposure time. The relevant temporal window will depend on the agent, route and duration of administration, systemic exposure, elimination kinetics, active metabolites where applicable, and the extent and persistence of tumour exposure. Accordingly, early and delayed sampling points should be anchored, where possible, to clinically relevant exposure profiles rather than transferred unchanged across drugs or tumour types. In multi-agent protocols such as CHOP, attribution is more complex because sequential or overlapping drug exposures may generate distinct or interacting stress responses; mechanistic studies should therefore examine individual agents or explicitly account for treatment sequence when interpreting temporal effects.

First, temporal precedence and association should be established. In dog-derived cancer systems exposed to pharmacologically justified concentrations and schedules of clinically relevant cytotoxic agents, early stress-signalling readouts should reproducibly precede delayed miRNA changes. Candidate early readouts may include YAP localisation or phosphorylation, p53 pathway activation, and stress-activated kinase signalling (Bao et al., 2011; Canovas & Nebreda, 2021; Liu et al., 2021; Lv & Zhou, 2023; Raj & Bam, 2019; Zhang et al., 2009). Later readouts may include mature miRNAs implicated in canine lymphoma or osteosarcoma biology, such as miR-21, miR-34a, or selected 14q32 miRNAs (Craig et al., 2019; Elshafie et al., 2023; Elshafie et al., 2024; Gourbault & Llobat, 2020; Lopez et al., 2018; Ludwig et al., 2024), measured as candidate network components rather than predefined markers of sensitivity or resistance. The exact time windows should be empirically calibrated rather than imposed as universal thresholds. Such calibration should consider drug-specific exposure and pharmacodynamic timing, because identical nominal sampling times may represent different biological phases for different agents. Beyond temporal ordering, variation in the early response should be tested for reproducible association with the later miRNA response. These molecular measurements should also be paired with orthogonal cell-fate readouts, including early apoptosis or DNA-damage assays and later viability or outgrowth assays. Such findings would support temporal association but would not, by themselves, establish mechanistic dependence or causal coupling. Because tumour-cell intrinsic responses may be modified by the tumour microenvironment, time-course experiments should, where feasible, distinguish neoplastic-cell readouts from signals arising in immune or stromal compartments. Complementary ex vivo, co-culture, or cell-type-resolved tissue analyses would help determine whether observed temporal changes are intrinsic to tumour cells or are shaped by microenvironmental interactions. The evidence structure and minimal tests for the proposed hypothesis are summarised in Table 1.

**Table 1:** Evidence status, uncertainties, and minimal tests for the proposed temporal coupling hypothesis.

Model component	Current evidence status	Key uncertainty	Minimal test	Interpretation if not supported
Early stress-signalling window	Biologically plausible. Hippo–YAP, p53, and p38/JNK pathways are relevant to cancer stress responses; direct canine support is stronger for p53 pathway activity than for chemotherapy-induced Hippo–YAP kinetics.	Whether canine tumour cells show reproducible early stress-signalling kinetics under drug-specific, pharmacologically relevant exposure conditions.	Perform drug-specific time-course studies in dog-derived lymphoma and osteosarcoma systems using pharmacologically justified concentrations and exposure schedules; measure YAP localisation or phosphorylation, p53 pathway activity, and stress-activated kinase signalling after treatment.	Absence of reproducible early stress signalling would weaken the model and require reconsideration of the proposed initiating phase.
Delayed miRNA-regulatory window	Supported as a candidate regulatory layer. Canine lymphoma and osteosarcoma studies link miRNA patterns, including miR-21, miR-34a, and 14q32-associated miRNAs, with tumour biology, prognosis, relapse, or aggressive behaviour. These associations do not establish a binary sensitivity–resistance role for individual miRNAs.	Whether post-treatment miRNA changes emerge in a reproducible temporal order and whether changes in mature miRNA abundance reflect altered biogenesis or other transcriptional/post-transcriptional processes.	Measure mature miRNAs across early and delayed post-treatment time points; where altered biogenesis is proposed, quantify pri-, pre-, and mature miRNA species in parallel, ideally paired with cell-fate readouts.	Inconsistent mature-miRNA changes would weaken the proposed delayed regulatory phase; mature-miRNA changes alone would not establish altered biogenesis.
Tumour microenvironment context	Biologically relevant but not integrated into the temporal hypothesis experimentally. Canine DLBCL and osteosarcoma show immune and stromal heterogeneity associated with tumour biology or outcome (Ammons et al., 2024; Aresu et al., 2019; Withers et al., 2019).	Whether immune or stromal compartments modify early stress signalling, delayed miRNA responses, or both, and whether bulk-tissue signals originate from neoplastic versus non-neoplastic cells.	Where feasible, complement tumour-cell assays with ex vivo, co-culture, or cell-type-resolved tissue analyses across matched time points, including relevant immune or stromal readouts.	Failure to account for microenvironmental contributions could confound attribution of temporal changes to tumour-cell-intrinsic mechanisms; absence of an effect in one model would not exclude context-dependent effects in vivo.
Temporal precedence and association	Conceptual basis of the hypothesis, but not yet established in canine cancer systems.	Whether early stress readouts reproducibly precede and are associated with later miRNA changes in canine tumour systems.	Use repeated time-course experiments with predefined sampling windows, then test associations between early stress readouts, later miRNA responses, and cell-fate outcomes.	Lack of reproducible temporal ordering or association would argue against the proposed biphasic relationship.
Hippo–YAP dependence of the miRNA response	Plausible but unvalidated in canine systems. Mammalian evidence supports Hippo–YAP regulation of miRNA biology, but canine chemotherapy-induced dependence has not been demonstrated.	Whether the delayed miRNA response depends on Hippo–YAP activity in canine tumour cells.	Perturb upstream Hippo activity or YAP localisation/function, verify pathway modulation, and measure delayed miRNA processing and mature miRNA abundance after chemotherapy.	If the delayed miRNA response is unchanged despite verified Hippo–YAP perturbation, mechanistic dependence on this axis would not be supported.
Candidate YAP–DDX17–Microprocessor-mediated coupling	Mechanistically plausible but not dog-validated. Human-cell studies support a YAP–DDX17/Microprocessor link, but this cannot be assumed in canine tumours.	Whether treatment-sensitive YAP–DDX17 interaction mediates downstream pri-to-pre-miRNA processing in canine tumour cells.	Demonstrate YAP–DDX17 interaction before and after cytotoxic exposure, perturb the proposed interaction, and measure pri-/pre-/mature miRNA processing; rescue or complementary perturbation should be used where feasible.	Interaction without a downstream processing effect would not establish causal coupling; absence of interaction or mediation would argue against this candidate mechanism but would not by itself refute the broader temporal hypothesis.
Clinical translation	Premature as a primary claim. Longitudinal clinical sampling in canine DLBCL could eventually test whether early stress readouts and delayed miRNA patterns associate with remission duration.	Whether cellular temporal patterns are detectable in clinically accessible samples and whether they add information beyond established clinical and pathological variables.	After cellular and assay validation, perform longitudinal studies with defined sampling times, standardised treatment context, and remission or relapse endpoints.	A negative clinical association would weaken biomarker utility, even if some cellular mechanisms remain biologically valid.

**Abbreviations:** DDX17, DEAD-box helicase 17; DLBCL, diffuse large B-cell lymphoma; JNK, c-Jun N-terminal kinase; miRNA, microRNA; YAP, Yes-associated protein.

Evidence status is interpreted according to the four-level hierarchy defined in Section 2.

Second, mechanistic dependence should be tested by perturbation. If Hippo–YAP activity is proposed to influence the later miRNA response, perturbing this axis should alter that response after chemotherapy. This could be tested by manipulating upstream Hippo activity, YAP localisation, or YAP function, followed by paired measurement of stress markers, pri-/pre-/mature miRNA species, and cell-fate outcomes. These RNA species should be interpreted as distinct readouts: mature-miRNA abundance reflects the final measured pool, whereas changes across pri-miRNA and pre-miRNA provide more direct information about precursor processing. Where assay performance permits, relationships between pri-, pre-, and mature species may support inference about processing efficiency, but should not be treated as standalone proof of altered biogenesis. Such experiments should verify that the intended pathway modulation was achieved and, where feasible, use complementary perturbations to reduce off-target interpretation. A reproducible downstream change following verified upstream perturbation would support mechanistic dependence, but would not by itself establish that the proposed YAP–DDX17 route mediates the effect. Conversely, if the delayed miRNA response is unchanged despite verified Hippo–YAP perturbation, dependence on this axis would not be supported.

If YAP–DDX17–Microprocessor is pursued as one candidate bridge, causal coupling through this specific route would require evidence beyond temporal ordering and upstream perturbation. Human-cell studies support a mechanistic link between Hippo signalling, YAP, DDX17/p72, and Microprocessor activity (Mori et al., 2014; Wu, 2020; Xu et al., 2022; Yu & Guan, 2014), but this cannot be assumed in canine tumours. The first requirement is to demonstrate a basal YAP–DDX17 interaction in canine lymphoma or osteosarcoma cells and determine whether that interaction changes after cytotoxic treatment. The second is to show that perturbation of the proposed interaction alters pri-to-pre-miRNA processing and the downstream miRNA response. Where experimentally feasible, rescue or complementary perturbation experiments would further strengthen causal attribution. Direct YAP–DDX17 interaction without a corresponding downstream processing effect would establish physical association but would not be sufficient to demonstrate causal coupling. Accordingly, a change in mature miRNA abundance without evidence of altered pri-to-pre-miRNA processing would not support a Microprocessor-mediated explanation. Failure to validate this route would argue against YAP–DDX17–Microprocessor as the relevant mediator, but would not by itself refute the broader temporal hypothesis.

Finally, clinical translation should be treated as a downstream test, not the starting point. Longitudinal sampling in canine diffuse large B-cell lymphoma could eventually ask whether early stress readouts and later miRNA patterns are associated with duration of first remission. However, proposed liquid-biopsy markers, including exosomal YAP-related measurements, require analytical validation before they can support clinical claims. A negative clinical association would not necessarily refute every cellular aspect of the model, but it would weaken its value as a biomarker strategy.

## 6 Discussion: implications, boundaries, and future directions

The immediate implication of this Opinion Paper is methodological rather than therapeutic. If acquired chemoresistance is partly shaped by the timing of post-treatment responses, then studies in these canine tumour contexts should not rely only on baseline samples, relapse samples, or drug-resistant cell lines selected after prolonged exposure. These designs remain useful, but they are poorly suited to detecting early treatment-induced transitions. A stronger approach would include defined early and delayed post-treatment time points in canine cell lines, ex vivo tumour material, or carefully selected clinical studies, with paired measurements of stress signalling, miRNA expression, and cell-fate outcome.

Pharmacokinetic and treatment-context heterogeneity are therefore integral to the temporal hypothesis rather than peripheral technical considerations. A nominal 6-, 24-, or 48-hour interval may have different biological meaning after different cytotoxic agents, depending on systemic and tumour exposure, drug persistence, active metabolites, and treatment sequence. This is particularly relevant to canine DLBCL, where CHOP protocols expose tumours to multiple agents with distinct pharmacological profiles, and to osteosarcoma, where the relevant temporal response may differ between drugs used in adjuvant treatment. Experimental time courses should therefore be drug-specific and exposure-informed, and clinical sampling should be interpreted relative to the actual treatment schedule rather than by clock time alone.

Canine diffuse large B-cell lymphoma is a logical first clinical context for this work. It has a clear therapeutic structure, high initial response rates to CHOP-based protocols, and clinically meaningful variation in remission duration and relapse (Childress et al., 2018; Davies et al., 2018; Hawkes et al., 2024; Vos et al., 2019). Osteosarcoma is also relevant, particularly for studying treatment failure, invasive behaviour, and tumour-suppressive miRNAs in dog-derived models (Berg et al., 1997; Gourbault & Llobat, 2020; Lopez et al., 2018; Ludwig et al., 2024; Selvarajah & Kirpensteijn, 2010). However, the model should not be generalised across all canine cancers without direct testing. Different tumour types, drugs, genetic backgrounds, and treatment schedules may produce different temporal patterns. Accordingly, DLBCL and osteosarcoma should be evaluated independently, and concordant findings across them would strengthen the broader relevance of the framework but would not establish a universal canine cancer mechanism. Such heterogeneity is inherent to spontaneous canine cancers. Any useful temporal model must therefore remain robust to inter-individual variability, treatment context, and pre-existing resistant subclones.

The boundaries of the model are equally important. The YAP–DDX17–Microprocessor axis is only one candidate mechanism and is not a validated canine pathway. The broader temporal hypothesis does not require this specific axis to be operative. The miRNA examples used in this article should not be interpreted as a binary sensitivity–resistance axis. miR-34a, selected 14q32 miRNAs, and miR-21 are included as illustrative candidates because canine studies have linked them to tumour biology, prognosis, relapse, or aggressive behaviour (Craig et al., 2019; Elshafie et al., 2023; Elshafie et al., 2024; Gourbault & Llobat, 2020; Lopez et al., 2018; Ludwig et al., 2024), not because current evidence establishes any of them as directionally specific markers of chemotherapy sensitivity or resistance. Additional canine-specific evidence comes from mammary tumours, in which miR-18a, miR-18b, and miR-21 were upregulated in malignant tumours compared with control tissues, whereas miR-146b was downregulated in both benign and malignant tumours (Abbate et al., 2022). A subsequent study linked miR-21 upregulation with increased IL-6 and TNF-α expression and with higher proliferative activity assessed by Ki67 in canine mammary carcinomas (Abbate et al., 2023). These cross-sectional findings broaden the evidence for biologically relevant miRNA dysregulation in canine tumours but do not establish chemotherapy-induced temporal regulation or chemoresistance. Individual miRNAs can regulate multiple targets, and their functional effects may vary with tumour type, treatment, and cellular state. miRNA findings should therefore be interpreted as context-dependent regulatory patterns rather than as isolated binary switches. Similarly, extracellular vesicle or liquid-biopsy readouts may become useful in future longitudinal studies, but they should not be treated as validated biomarkers until analytical performance, biological source, and association with outcome have been established.

The tumour microenvironment is another important source of biological context that is not captured by a purely tumour-cell-intrinsic model. In canine DLBCL, transcriptional studies have identified immune and inflammatory programmes, including T-cell-, macrophage-, and immune-checkpoint-related signals, associated with clinical outcome (Aresu et al., 2019). Canine osteosarcoma also contains a heterogeneous immune microenvironment, including multiple tumour-associated macrophage populations and predicted dendritic-cell/T-cell interactions (Ammons et al., 2024), and macrophage infiltration has been associated with disease-free interval in dogs treated with amputation and adjuvant carboplatin (Withers et al., 2019). These observations support the biological relevance of the microenvironment but do not establish that it drives the proposed post-chemotherapy temporal sequence. Cytokine signalling, immune-cell composition, stromal interactions, hypoxia, and extracellular-vesicle exchange could modify either the early stress response or later miRNA regulation and may also contribute signals detected in bulk tumour samples. Future temporal studies should therefore distinguish tumour-cell-intrinsic changes from microenvironment-derived responses wherever experimentally feasible.

Cross-species time-resolved evidence provides limited precedent for this type of temporal design. In K562 chronic myeloid leukaemia cells, imatinib exposure was associated with an early Hippo-related transcript response at 12 h followed by delayed upregulation of Hippo-associated miRNAs at 48 h (Akbari-Ardabili et al., 2026). However, these findings were correlative, were obtained in a human cell-line model, and were not reproduced as a uniform Hippo–YAP transcriptomic response in primary CD34⁺ CML samples. They therefore support the feasibility of testing temporally separated Hippo- and miRNA-related responses, but not the existence of the proposed mechanism in canine tumours.

This Opinion Paper does not argue that temporal coupling is the sole explanation for chemoresistance. Resistant disease may arise through clonal selection, pre-existing heterogeneity, drug efflux, impaired apoptosis, altered DNA repair, immune and stromal microenvironmental influences, pharmacokinetic variation, or treatment heterogeneity. The proposed model is useful only if it helps organise these factors into testable temporal questions. Its near-term value is therefore not clinical prediction, but disciplined hypothesis generation: identifying when treatment response begins to fail, which regulatory transitions are involved, and which claims can be supported, revised, or rejected in canine systems.

## Funding

This research did not receive any specific grant from funding agencies in the public, commercial, or not-for-profit sectors.

## Ethics statement

This article did not involve new animal experiments, human participants, or privately owned animal data. Therefore, ethics approval was not required.

## Declaration of generative AI and AI-assisted technologies in the manuscript preparation process

During the preparation and revision of this work, the author used ChatGPT (OpenAI) for language refinement and editorial organisation. After using this tool, the author reviewed and edited the content as needed, independently verified the scientific claims and references, and takes full responsibility for the content of the published article.

## Declaration of competing interest

The author declares that they have no known competing financial interests or personal relationships that could have appeared to influence the work reported in this paper.

## Data Availability

No original datasets were generated or analysed for this Opinion Paper. All literature discussed in the manuscript is available from the cited sources.
